# An Alternative Approach for Selective Lung Ventilation

**DOI:** 10.7759/cureus.38126

**Published:** 2023-04-25

**Authors:** Husam Alsamman, An Bui, Justin Howard, Muhammad Helwany, Ramneek Maan, Kanaan Samer

**Affiliations:** 1 Anesthesiology, Riverside Community Hospital, Riverside, USA; 2 Thoracic Surgery, Riverside Community Hospital, Riverside, USA

**Keywords:** icu, thoracic surgery, uniblockers, single lung ventilation, double lumen tube

## Abstract

Single lung ventilation (SLV) with the double lumen tube (DLT) has been an effective process for providing surgical exposure in the thoracic cavity and has been applied effectively in the operating room. SLV also aids in protecting a healthy lung from the ill effects of fluid from an unhealthy lung, which may be blood, lavage fluid, or malignant or purulent secretions. Ensure correct placement, which is required and confirmed by a fiberoptic bronchoscope (FOB).

The use of the DLT has been proven to be effective, but it has its challenges and drawbacks. This article proposes an alternative technique to the DLT in SLV without the use of a FOB. We have implemented this technique in 14 cases, however, we would like to discuss two challenging cases that have highlighted the advantages of this new technique.

## Introduction

Lung separation and selective lung ventilation are crucial elements in the management of patients undergoing thoracic and thoracoabdominal surgery, as well as in the care of ICU patients suffering from lower airway bleeding, traumatic airway disruptions, major lung leaks, and other complications of mechanical ventilation.

The use of double-lumen tubes (DLT) and "uniblockers" is an effective method for lung isolation to achieve selective lung ventilation. However, their placement and positioning may present several challenges, including loss of airway or airway injuries [1]. Some of these challenges may be worsened in the pediatric population: previously intubated patients, known difficult airways, left or right main bronchus stenosis, and lower airway hemorrhage. In addition, malpositioning of the DLT can lead to life-threatening consequences such as severely impaired ventilation leading to hypoxia, gas trapping, tension pneumothorax, cross-contamination of lung contents, and interference with surgical procedures [2]. Another challenge that is associated with the usage of the DLT is the frequency of malpositioning after direct laryngoscopy and the necessity of the fiberoptic bronchoscope (FOB). This may be due to the lack of guidelines in the literature that help select the appropriate size and insertion depth for the DLT [3]. Additionally, distorted anatomy may complicate the insertion process. Studies have shown that measuring the tracheal diameter through CT studies has aided in choosing the appropriate sizing, however, this may increase hospital costs and unnecessary exposure to radiation [4].

To address these difficulties, we proposed and successfully utilized an alternative approach to achieve lung separation and selective lung ventilation that could potentially be life-saving in the aforementioned difficult cases. Some advantages of this technique include its flexibility, ease, and availability, especially in low-resource settings with scarce availability of DLT and FOB.

## Materials and methods

**Figure 1:**
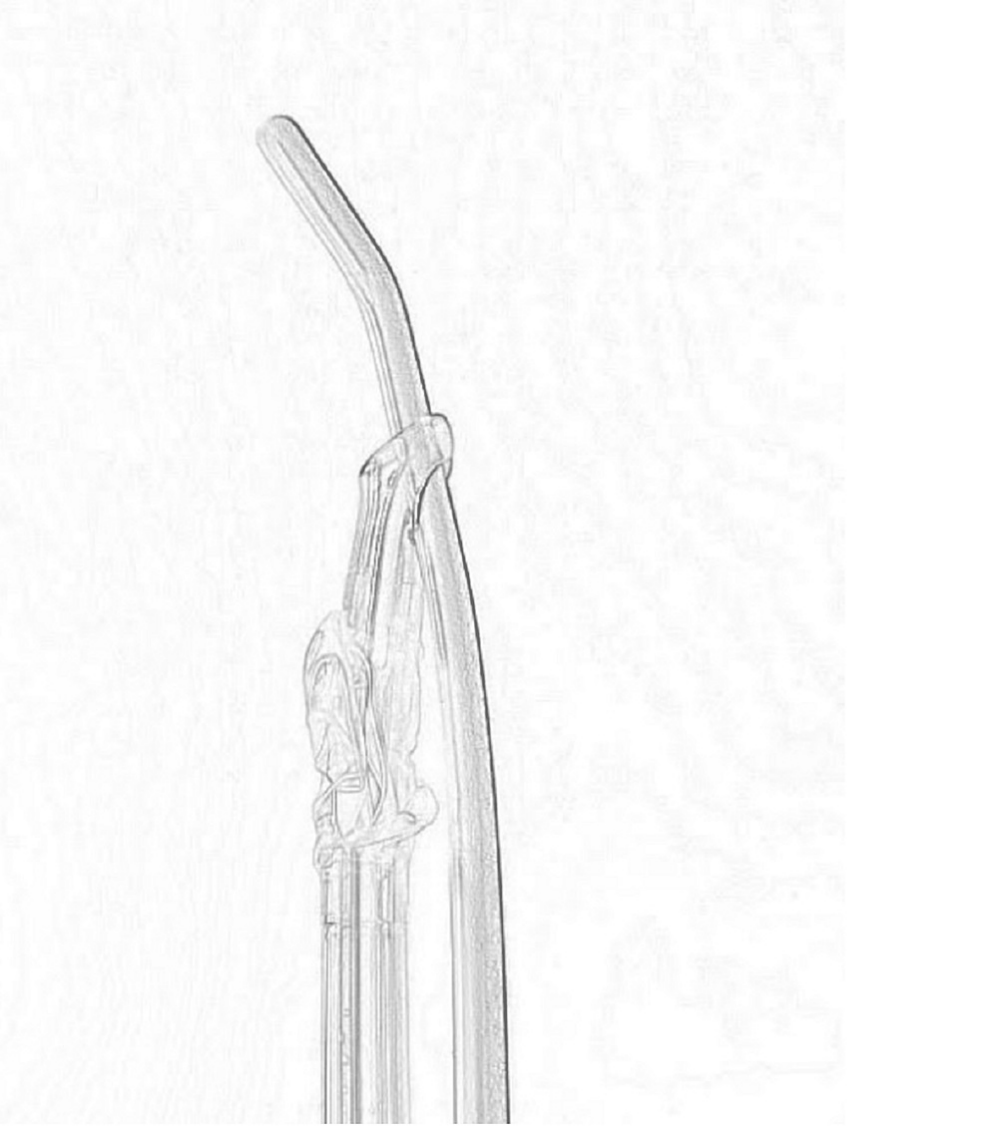
Advancement of the first endotracheal tube through the bougie using the Murphy eye

The planned action was to insert two endotracheal tubes (ETT) in the trachea instead of one single DLT. It begins with direct laryngoscopy and the initial insertion of a single-use plastic bougie. Once the bougie is in, the first ETT is guided into the trachea using the Murphy eye (Figure 1).

**Figure 2:**
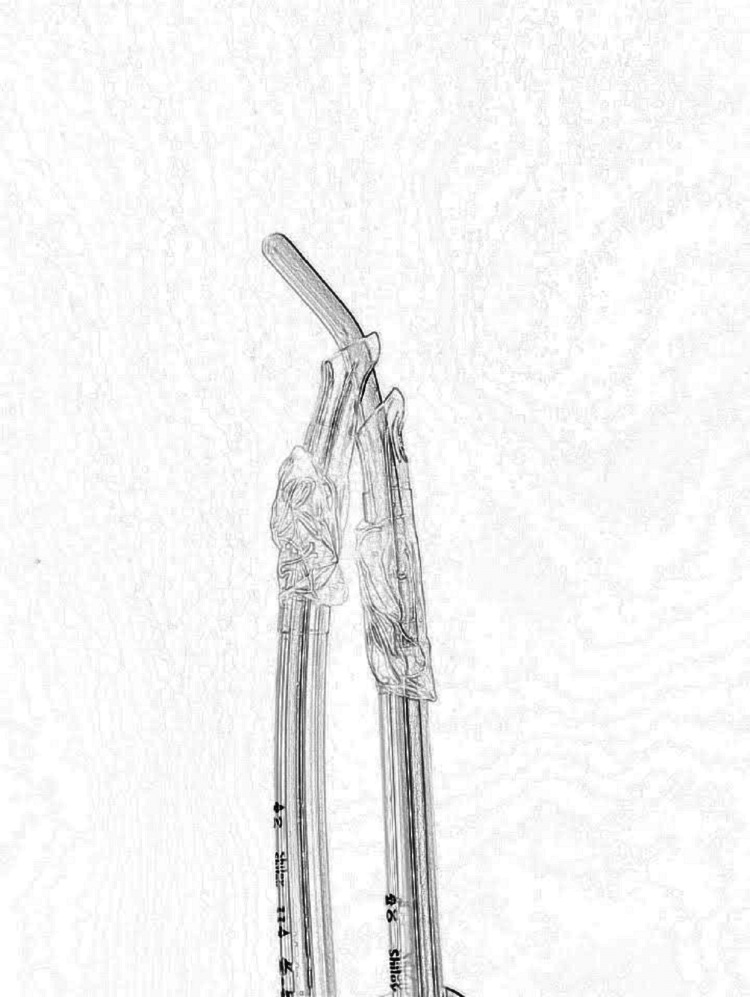
Insertion of the second endotracheal tube guided by the bougie in traditional fashion

The second endotracheal tube is then inserted in the traditional fashion on top of the first endotracheal tube (Figure 2).

The bougie is then removed, leaving behind the two endotracheal tubes in the trachea, and then used again to guide one ETT in the right mainstem bronchus with its straight end and guide the second ETT in the left mainstem bronchus with its curved end.

**Figure 3:**
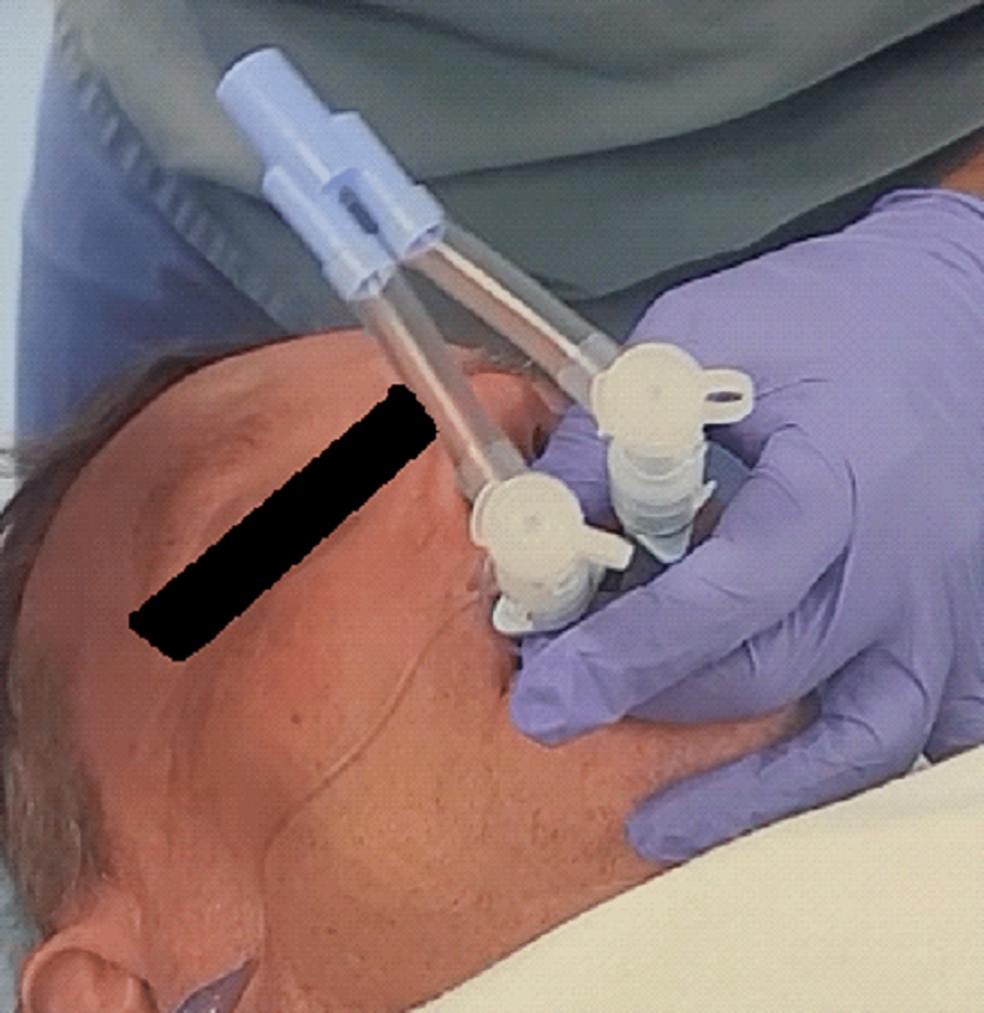
Traditional adaptors for the double lumen tube used on two individual endotracheal tubes

The results are two individual endotracheal tubes that can be attached to one or two ventilators using the adaptors traditionally used for the double-lumen tube (Figure 3).

**Figure 4:**
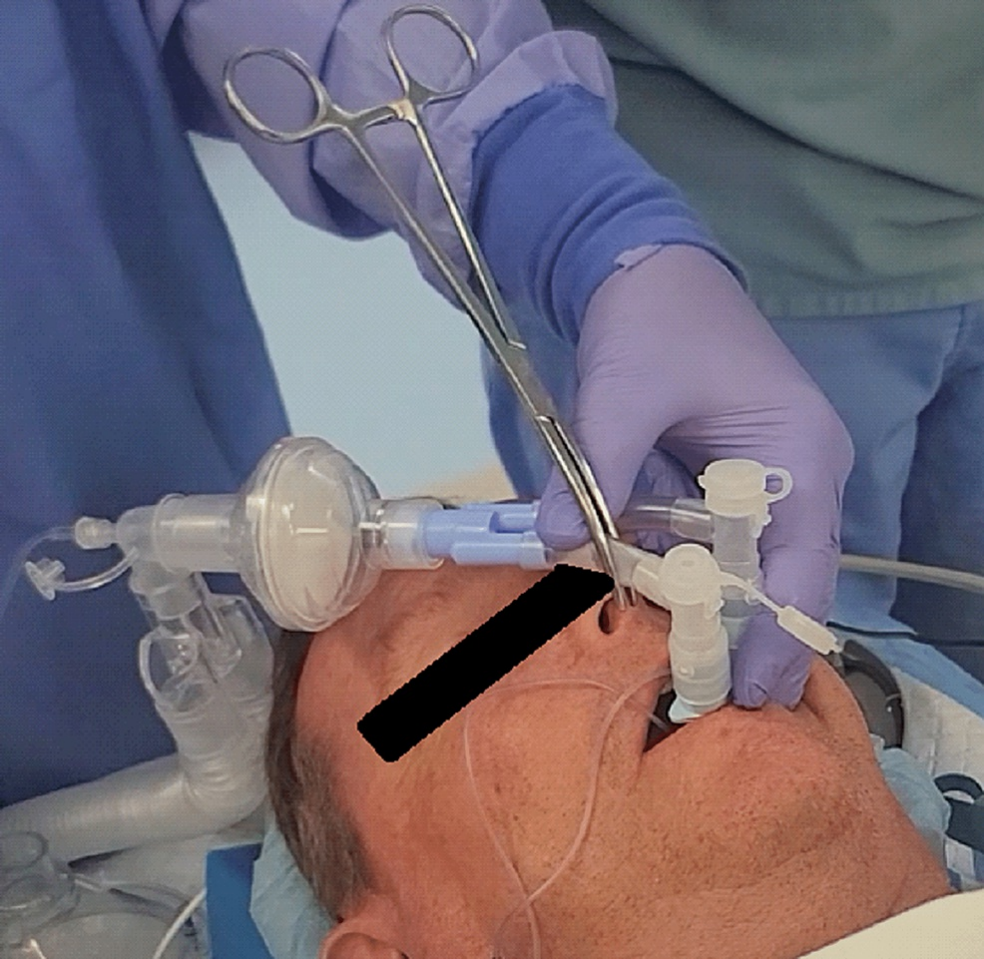
Right lung isolation through clamping of the right mainstem bronchus endotracheal tube

To achieve left or right lung isolation, the respective endotracheal tubes can be clamped in a traditional fashion (Figure 4).

If the airway is already intubated, then an endotracheal tube exchanger could be used instead of the bougie through the already existing endotracheal tube in order to blindly exchange the single endotracheal tube for two endotracheal tubes as described above.

After insertion, the two tubes are connected to the double-lumen tube adapter. Verification of correct positioning with the fiberoptic bronchoscopy is helpful but not necessary in this technique. Regular-length endotracheal tubes are long enough for most female, and short male patients, but mighty long tubes (MLT) are needed for tall females and for most adult male patients. To isolate the lungs, both ETT cuffs are inflated with 10 ml of air, and then the appropriate tracheal tube is clamped.

Approval was obtained from the hospital research ethics committee to use this new technique on consenting patients as part of our investigation. Signed consent documentation was done bedside. The cases were all performed at a community hospital.

## Results

We would like to highlight some of the cases we handled to demonstrate the unique advantage offered by our new technique.

Case 1

A 56-year-old male diagnosed with class 1 obesity and a right upper lobe mass presented for a robotic right upper lobe resection. On preoperative evaluation, he screened positive for sleep apnea and had clinical features suggestive of possible difficult intubation. The team planned for direct laryngoscopy with a videoscope as a backup.

The principal investigator (PI) performed a direct laryngoscopy. The patient was a grade IIIB on the Cormack-Lehane scoring criteria. A bougie was successfully placed into the airway, and then two 6.5-mm armored endotracheal tubes were placed over the bougie in the manner described earlier. The patient was intubated with a single attempt, with a total intubation time of three minutes. The heart rate and blood pressure were within 20% of baseline throughout the intubation process.

Successful placement of both ETTs into their respective bronchi was confirmed during manual bag ventilation by clamping off one ETT and auscultating the patient’s lung fields. The patient’s oxygen saturation was 95-97% during supine two-lung ventilation.

When the patient was placed into the left lateral decubitus position for the right upper lobectomy (RUL) resection and the right-sided ETT was clamped for right lung isolation, the patient began to desaturate to the low 80% range before surgery began. We converted back to two-lung ventilation with successful ventilation and oxygenation of the patient back to over 95%. Once stable, we confirmed the correct positioning of both ETTs during fiberoptic evaluation. However, when we re-attempted right lung isolation by clamping the right-sided ETT, the patient began to desaturate back to 80-82% despite the standard maneuvers to combat one-lung ventilation (OLV) desaturation. We decided to unclamp the right ETT and push it past the RUL bronchus takeoff so that only the right middle and lower lobes were ventilated. This improved the patient’s oxygenation back to 95-97%, allowing surgery to proceed. We could now ventilate the right middle lobe (RML) and right lower lobe (RLL) with a small tidal volume during surgery as well as provide partial right lung isolation to facilitate the surgery. The RUL resection case was successfully performed, and the patient’s two endotracheal tubes were easily extubated at the end of the case. The patient was moved to the post-anesthesia care unit and ultimately to the hospital floor with no problems.

A post-operative chest X-ray did not show any left-sided lung abnormalities that could explain the poor oxygenation during one-lung ventilation with the left-sided ETT. Patient evaluation on postoperative day 1 on the floor was unremarkable, with no other anesthetic concerns. He was discharged from the hospital on POD 4.

Case 2

A 54-year-old female diagnosed with mild asthma and tobacco dependence (10 pack years) presented to the ER with shortness of breath exacerbated when lying in a supine position and difficulty speaking. The patient could whisper fragmented words but was unable to speak loudly or verbalize short sentences. On examination, the patient was dyspneic with no obvious deformities or lesions. Vital signs include 98 °F, 95 beats per minute, blood pressure 140/80, and saturating at 94% on a 2-liter nasal cannula. Pulmonary function tests were not obtained.

**Figure 5:**
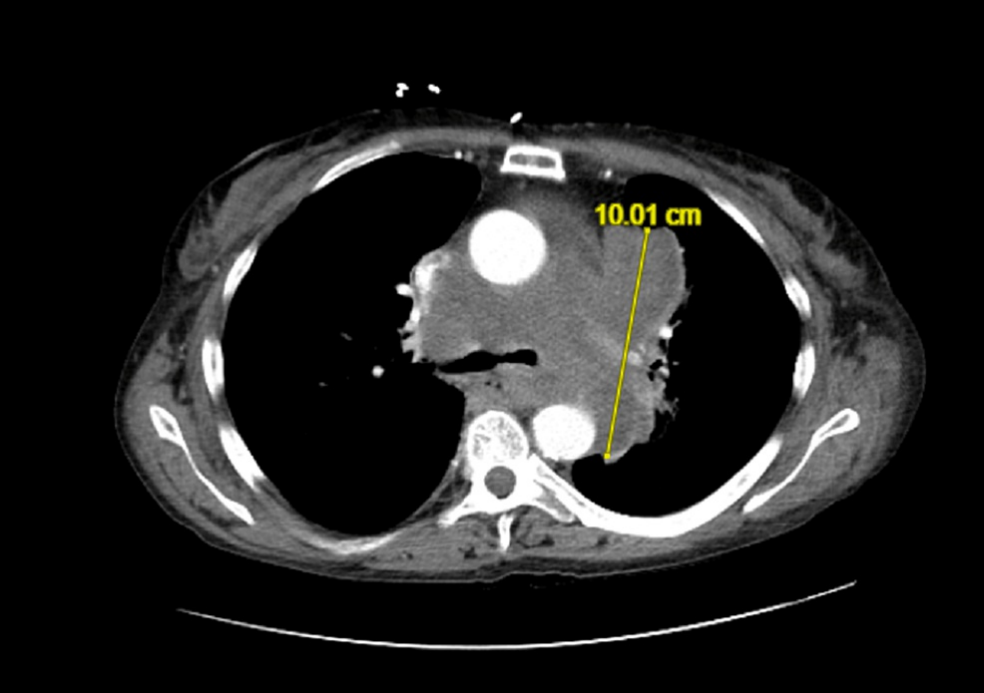
CT chest with IV contrast

A CT chest with IV contrast revealed the following: a large lobulated conglomerate soft tissue mass encasing the trachea and bronchi, the pulmonary arteries, and partially encircling the aorta. In summation, the mass measured approximately 10 cm AP by 11.5 cm transverse. The soft tissue mass extended along the left hilum and partially encased the left long proximal airways. Mild narrowing of the pulmonary arteries and airways, greater on the left. There is a right posterolateral displacement of the upper thoracic esophagus. Compression and lateral displacement of the superior vena cava, which remains patent (Figure 5). The narrowest point was at the right main bronchus, measuring 0.15 cm.

The interventional radiology team was consulted but concluded that a biopsy was not feasible and referred the patient to thoracic surgery due to the critical location of the mass and the patient's worsening status. The surgeon consulted the anesthesia service for evaluation and scheduled the patient for pericardial window, left VATS for a biopsy, and lymph node biopsy with one lung ventilation. The patient was evaluated by two different anesthesiologists, who both concluded that attempted lung isolation in this patient would be too high-risk and extremely difficult. The case was then referred to the cardiac anesthesia team to evaluate whether she could proceed with surgery with the assistance of femoro-femoral ECMO or partial cardiopulmonary bypass.

This patient posed several additional anesthetic challenges as she was extremely frail with a BMI of 14, had limited mouth opening, and had limited neck range of motion due to a history of cervical fusion. Because the anterior mediastinal tumor caused the narrowing of the airway, the goal of management was to maintain spontaneous respiration/muscle tone to prevent the complete, irreversible collapse of the airway. In addition, the need for heparinization with the use of ECMO increased the risk of massive bleeding, frailty, and the fear of the inability to wean the patient from ECMO with the tumor still compromising the airway made ECMO or partial cardiopulmonary bypass far less favorable over other choices. 

The patient was brought to the operating room and placed on standard ASA monitors. Given the symptoms, the size and location of the mediastinal mass, and the airway assessment, we decided to completely avoid paralytics to maintain chest wall muscle tone and prevent airway collapse. Two milligrams of IV midazolam were administered, and a transtracheal injection with 2% lidocaine was performed with a fine needle to anesthetize the vocal cords and lower airway to facilitate intubation without the assistance of muscle relaxation. The patient then underwent induction using 40 mg of propofol and 100 mcg of fentanyl in combination with sevoflurane inhalation. A direct laryngoscopy was performed, and a tracheal intubation Bougie was easily inserted into the airway as described in the above pictures to enable us to insert two endotracheal tubes; a 5 mm ETT through its Murphy's eye directed toward the left main bronchus, and a 6 mm ETT through the lumen of the tube directed towards the right main bronchus. Pre-induction vitals were: 90 beats per minute, blood pressure of 128/86, and saturation at 94% on a 10-liter face mask. Post-induction vitals were: 98 beats per minute; blood pressure 158/90; saturation at 98% on 100% oxygen. The total intubation time was four minutes with one attempt.

Lung isolation was successfully achieved using the two single-lumen tubes. End-tidal CO_2_ was noted with the lung separation confirmed visually and via auscultation and the patient was placed on mechanical ventilation. The use of FOB for further confirmation of lung isolation was considered but intentionally avoided in this case to minimize distal airway manipulation. In the presence of such an invasive tumor and the absence of deep muscle relaxation, we wanted to avoid irritation to the distal airways, which could potentially result in coughing or bucking, causing airway collapse.

Oxygen saturation was maintained at greater than 90% throughout induction, intubation, and one-lung ventilation. The surgery was successful with a duration of 79 minutes, and the surgeon was completely satisfied with the left lung isolation and one-lung ventilation. A pericardial window was performed, and the mediastinal mass and lymph nodes were successfully biopsied. An intercostal block for post-op pain control was performed at the end of surgery before extubating the patient awake in the sitting position. The patient spent an hour in post-anesthesia care unit (PACU) and was then transferred to ICU for observation, where she remained extubated awaiting the tissue diagnosis. Biopsies later revealed a diagnosis of advanced small cell carcinoma.

## Discussion

The use of a double-lumen tube is the current standard of care for lung isolation, however, it has several drawbacks.

Compared to a single-lumen ETT, the DLT is longer, stiffer, and has a larger bore, which may create difficulty during tracheal intubation, especially in difficult airways. The DLT is also prone to dislodging during positioning of the patient from supine to left/right lateral decubitus. Additionally, there are case reports of successful lateral insertion of DLTs in a conventional way in low-resource settings [5].

The proposed new technique utilized in our center with success in elective and semi-elective cases may help address the issues faced with using the DLT. The insertion technique is simple and could be performed with basic airway management skills on any easy or difficult airway that needs lung separation, including "trached" or intubated airways. We have highlighted two cases that have demonstrated successful lung isolation with no adverse consequences. O_2_ saturation remained >90% in all cases. Compared to the DLT, there is less risk of losing a secure airway in a patient because we are initiating tracheal intubation with a rescue bougie to place the two endotracheal tubes. The familiarity of using a single-lumen ETT and the bougie also aids in facilitating tracheal intubation in a known difficult airway and does not require confirmation of correct placement with a fiberoptic bronchoscope. Although the fiberoptic bronchoscope is not mandatory, in the first case described above, we used it to guide or correct positioning immediately after placement as well as after patient positioning. We found that the tubes remained secure in the correct position and did not misalign. Re-inflating the nondependent lung as well as double lung ventilation also posed no issues.

One of the unique advantages offered by this new technique is described in the first case of a patient undergoing an RUL resection who was not able to tolerate OLV. When the right tracheal tube was clamped, the patient would desaturate down to a low of 80%. If a DLT had been used, the surgery would typically be intermittently paused to initiate double lung ventilation, and surgery would resume intermittently after the saturation improved. However, with the new technique, we were able to partially ventilate the RML and RLL while keeping the RUL deflated, which however, prevent severe hypoxia and allowed the surgeon to continue to operate. As a counterargument, the surgery would have been salvaged by a routine DLT without converting to double lung ventilation, however, this would mean the surgeon would only be able to work intermittently during periods of apnea.

Another unique advantage is described in the second case in an unstable patient with a large thoracic mass and severe airway obstruction at baseline who would unlikely tolerate the placement and relatively large size of a double-lumen tube or be successfully weaned off ECMO without life-threatening complications. In addition, the patient's BMI of 14 is an indicator of severe frailty which poses a very high anesthetic risk. Placing a small-size uni-blocker in this case would have been extremely challenging which may force the anesthesiologist to ventilate through a single lumen tube with prolonged periods of apnea to facilitate the surgery. In our case, however, the surgery was of short duration. The use of the appropriately sized tracheal intubation bougie into both mainstem bronchi and two smaller endotracheal tubes facilitated the intubation and reduced the risk of losing or collapsing the distal airway while being able to achieve lung isolation.

This new technique has shown promise intraoperatively; however, further studies will need to be done. Future studies could also be extended to the ICU. Given its flexibility, ease compared to the DLT, and usefulness in low-resource settings with scarce availability of the DLT and FOB, an emergent airway can be established using the new technique that requires lung isolation or when lung isolation is needed when a FOB is not available. The technique is relatively quick and well tolerated and could be used in sick patients on high continuous positive airway pressure (C-PAP), high positive end-expiratory pressure (PEEP), or high FIO_2_ who might need quick ventilation. In those patients, ventilation could be achieved very quickly through the first inserted tube immediately after inflating its balloon before inserting the second endotracheal tube while keeping the bougie or tube exchanger in its place in the airway. In addition, this technique may also be favored in pediatric patients and in situations where a fiberoptic bronchoscope may not be available; however, the risks of airway injuries and airway edema are potential concerns with this technique.

Limitations

The insertion of two single-lumen ETTs may increase the risk of bleeding and airway trauma. In addition, there is also an increased risk of lung soiling if performed in a similar fashion as presented in case 1. For patients with anticipated difficult airways, the insertion technique may also be more challenging than the insertion of a DLT. In addition, we found that the length of the single-lumen ETTs may not be sufficient, especially in the taller population.

## Conclusions

This technique has shown promise in two challenging cases requiring single-lung ventilation. However, future clinical studies with larger sample sizes will be needed to determine the value of this technique. This technique could also theoretically be utilized in the ICU; however, we will need more data to support this. If this technique proves useful, it would be a powerful tool in the anesthesiologist's arsenal.
